# Effects of intramuscularly administered enrofloxacin on the susceptibility of commensal intestinal *Escherichia coli* in pigs (sus scrofa domestica)

**DOI:** 10.1186/s12917-017-1260-8

**Published:** 2017-12-04

**Authors:** Antje Römer, Gesine Scherz, Saskia Reupke, Jessica Meißner, Jürgen Wallmann, Manfred Kietzmann, Heike Kaspar

**Affiliations:** 1 Federal Office of Consumer Protection and Food Safety, Berlin, Germany; 2 University of Veterinary Medicine Hannover, Foundation, Institute of Pharmacology, Toxicology and Pharmacy, Hanover, Germany

**Keywords:** Parenteral administration, Enrofloxacin, Resistance, PFGE, *E. coli*, Macrorestriction

## Abstract

**Background:**

In the European Union, various fluoroquinolones are authorised for the treatment of food producing animals. Each administration poses an increased risk of development and spread of antimicrobial resistance. The aim of this study was to investigate the impact of parenteral administration of enrofloxacin on the prevalence of enrofloxacin and ciprofloxacin susceptibilities in the commensal intestinal *E. coli* population.

**Methods:**

*E. coli* isolates from faeces of twelve healthy pigs were included. Six pigs were administered enrofloxacin on day 1 to 3 and after two weeks for further three days. The other pigs formed the control group. MIC values were determined. Virulence and resistance genes were detected by PCR. Phylogenetic grouping was performed by PCR. Enrofloxacin and ciprofloxacin were analysed in sedimentation samples by HPLC.

**Results:**

Susceptibility shifts in commensal *E. coli* isolates were determined in both groups. Non-wildtype *E. coli* could be cultivated from two animals of the experimental group for the first time one week after the first administration and from one animal of the control group on day 28. The environmental load with enrofloxacin in sedimentation samples showed the highest amount between days one and five. The repeated parenteral administration of enrofloxacin to pigs resulted in rapidly increased MIC values (day 28: MIC up to 4 mg/L, day 35: MIC ≥ 32mg/L). *E. coli* populations of the control group in the same stable without direct contact to the experimental group were affected.

**Conclusion:**

The parenteral administration of enrofloxacin to piglets considerably reduced the number of the susceptible intestinal *E. coli* population which was replaced by *E. coli* strains with increased MIC values against enrofloxacin. Subsequently also pigs of the control were affected suggesting a transferability of strains from the experimental group through the environment to the control group especially as we could isolate the same PFGE strains from both pig groups and the environment.

## Background

Each administration of antibiotics in human and in veterinary medicine exerts a selective pressure and poses an increased risk of development and spread of antimicrobial resistance (AMR) [1]. This may affect not only zoonotic bacteria but also commensal bacteria in the intestine of food producing animals which are of special concern under public health aspects [2–4].

Fluoroquinolones are important antibiotics for the treatment of various bacterial infections in both humans and in animals [5], which have been categorised as “highest priority critically important antimicrobials” (HPCIA) for human and animal health [6, 7].

Fluoroquinolones exhibit a broad spectrum of antimicrobial activity against Gram-negative and Gram-positive bacteria. Their pharmacokinetic properties are characterised by a good bioavailability after oral as well as after parenteral application, an adequate distribution in tissue in association with high plasma levels and adequate renal clearance. Elimination is rapid via both urine and faeces [8–11]. In previous kinetic studies bioavailability and plasma concentrations were higher and absorption faster after intramuscular administration of fluoroquinolones [9, 12]. Wiuff et al. showed that i.m. administration of enrofloxacin in pigs resulted in a faster absorption and a more efficient distribution of enrofloxacin to plasma, lymph nodes and tissues of the intestine than p.o. administration [13]. But no significant differences in intestinal content concentrations between the administration routes were found except one measurement after 24 hours.The concentration dependent bactericidal activity of fluoroquinolones is based on inhibition of the target enzymes gyrase and topoisomerase IV [14, 15].

Diverse mechanisms may cause resistance to fluoroquinolones. Chromosomal mediated resistance develops by step-wise occurring point mutations in the target genes encoding for DNA gyrase (*gyrA* and *gyrB*) and topoisomerase IV (*parC* and *parE*). Increased number of mutations lead to an accumulating reduction of susceptibilities against fluoroquinolones [5, 16]. Other mechanisms are related to decreased intracellular accumulations of fluoroquinolones, e.g. those affecting the expression of outer membrane proteins or efflux pumps [17]. Plasmid mediated quinolone resistance (PMQR) in Enterobacteriaceae has been reported for 17 years [18–20]. But without additional chromosomally located mutations in the target genes plasmid mediated fluoroquinolone resistance results only in slight shifts of the particular minimal inhibitory concentration (MIC) [19, 21].

In the European Union, various fluoroquinolones are authorised for the treatment of food producing (cattle, pigs, poultry and rabbits) and companion animals [22]. Enrofloxacin was the first fluoroquinolone developed exclusively for the use in veterinary medicine. In food producing animals, main indications are the therapy of respiratory and gastrointestinal diseases. Enrofloxacin can be administered by subcutaneous injection to cattle and intramuscular injection to pigs or by preparations for oral use for cattle, pigs, turkeys and chicken. For piglets, numerous solutions for injections but only few preparations for oral administration are available. Therefore, administration of enrofloxacin to pigs seems mainly done by injection [23].

Resistance to enrofloxacin and other fluoroquinolones in intestinal commensal as well as pathogenic *E. coli* strains is low to moderate in European pig production systems although there are differences among countries and production types [4, 24]. As high-level resistance to fluoroquinolones commonly requires a mutational component, occurrence of fluoroquinolone-resistant *E. coli* strains is mainly influenced by the degree of its use [25] whereby some resistant *E. coli* strains may persist for at least two weeks after three-day treatment [26]. Resistance to one fluoroquinolone often result in resistance to all fluoroquinolones [27].

Via faeces and/or urine depending on the particular active ingredients, enrofloxacin and its antibiotic active metabolite ciprofloxacin are released into the surrounding. This can lead to carry-over or reconsumption of subtherapeutic concentrations by the animals or to the exposure of environmental bacteria to the residues [28, 29].

The objective of this study was to investigate the impact of parenteral administration of the fluoroquinolone enrofloxacin and its metabolite ciprofloxacin on the prevalence of non-wild type (N-WT)-*E. coli* isolates in the commensal intestinal *E. coli* population of pigs with and without direct administration of enrofloxacin. Additionally, the distribution of enro- and ciprofloxacin in sedimentation samples of dust from the stable was measured to determine the release of the active ingredients into the direct surrounding of the animals.

## Methods

### Animals

Prior to placement of the pigs onto the holding several pigs from different farms were screened for N-WT-*E. coli* isolates. Rectal swabs of 30 pigs per farm were enriched in 3 mL lysogeny broth (LB) [30] overnight at 37°C; 10 μL of the cell suspension were streaked onto Endo agar [31] supplemented with 0.125 mg/L, 0.25 mg/L and 4 mg/L enrofloxacin and onto Endo agar without supplementation as a control. Only pigs from farms without any coliform growth onto Endo agar with 0.125 mg/L, 0.25 mg/L and 4 mg/L enrofloxacin were assumed to be free of N-WT-*E. coli* and included in this study.

Twelve five to six weeks old pigs from a single farm were randomly divided into two groups of six pigs. Both pig groups were housed in straw bedded pens of 1.5 X 3 metres with a distance of 3 metres between both pens. Temperature was between 23°C and 24°C and the relative humidity was between 50% and 60%. Ventilation was done by a positive pressure system. In addition to daylight a lighting program of 10 hours of light was used simultaneously. The pigs were fed a commercial pig feed once daily and had free access to tap water.

Before the animals were placed in the pens, the whole stable was cleaned and disinfected according to good manufacturing practices (dry cleaning first, followed by high-pressure cleaning, afterwards disinfection with Venno® FF super (Menno Chemie Vertrieb GmbH, Norderstedt, Germany)). Environmental samples were taken by wiping with sterile boot swabs soaked in hypotonic sodium chloride. Thereafter each boot swab was enriched in 250 mL LB and streaked onto Endo agar supplemented with enrofloxacin (0.125 mg/L, 0.25 mg/L, 0.5 mg/L, 0.75 mg/L, 1 mg/L, 1.5mg/L and 4 mg/L) to screen for contamination with N-WT-*E. coli*. If, after cleaning and disinfection, no lactose-fermenting coliform growth occurred on these plates, the stable was assumed to be free of N-WT *E. coli* and pigs were placed in. All persons handling animals wore disposable protective gloves and appropriate protective clothing. All animals were clinically healthy during the entire experiment. Experimental procedures started after one week of acclimatisation to avoid excessive stress to the pigs.

The study was authorised by the Lower Saxony State Office for Consumer Protection and Food Safety, Niedersachsen, Germany (reference number 33.9-42502-04-11/0338).

### Study protocol

**Fig. 1:**
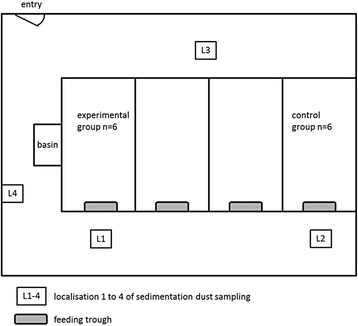
Schematic representation of the stable and the locations of sedimentation dust sampling

Six pigs (experimental group) were administered an intramuscular injection in the neck behind the ear with the recommended dosage of 2.5 mg/kg bodyweight (bw) enrofloxacin (Baytril® 10%) on day 1 to 3 and two weeks later (day 18–20). The other six pigs housed in a second pen in the same stable were used as control animals. Animals of both groups had no direct contact but the transmission of airborne particles or via vectors could not be excluded. To reduce carryover into the control group, feeding, administration of enrofloxacin and sampling of pigs from the control group were always done first. Rectal faecal samples were taken one day before the administration started and on the days 4, 11, 18, 21, 28, 35, 42 and 54. Additionally, Endo agar plates without supplementation of enrofloxacin were placed directly in front of the boxes close to the feeding trough of each group, between both boxes on the stall gangway and in the working area for animal keepers to isolate *E. coli* from the direct environment (Fig. 1).

### Isolation of *E. coli* from faeces and environment

Faecal samples were serially diluted in sodium chloride solution (0.9%), and 100 μL of each dilution were streaked onto Endo agar plates without supplementation of enrofloxacin and onto Endo agar plates supplemented with 0.125 mg/L enrofloxacin, a concentration equivalent to the epidemiological cut-off value [32]. Plates were incubated at 37°C for 20–24 hours. Ten lactose-fermenting coliform colonies per animal and plate were picked by a self-produced template, which localised ten fix points on every plate to avoid subjective selections. Isolates were confirmed as *E. coli* using LMX broth modified by Manafi and Ossmer [33] and indole reaction.

The animals were also screened for *E. coli* isolates with MIC values > 4 mg/L for enrofloxacin. After enrichment of each faecal sample in 9 mL LB-Bouillon at 37°C for 20–24 hours, 100 μL of the enriched cell suspension were streaked onto Endo agar plates supplemented with 4 mg/L enrofloxacin and incubated at 37°C for 20–24 hours as described by Scherz et al. [34].

During environmental sampling four Endo agar plates without supplementation of enrofloxacin were placed uncovered for 1.5 h at four defined locations of the stable.

If fewer than ten colonies were observed on a plate, all suitable colonies were used for further analysis. According to a previous study, the probability to isolate at least one colony of the most common strain in a faecal sample was more than 99% if five bacterial isolates were tested [35]. Another study showed that it would be sufficient to type ten isolates per sample to find all strains of verotoxigenic and non-type-specific *E. coli* in faeces samples with an 85% probability [36].

### Antimicrobial susceptibility testing

MIC values of ten *E. coli* colonies from each Endo agar plate supplemented with 0.125 mg/L enrofloxacin, one colony from each Endo agar plate with 4 mg/L enrofloxacin and maximum ten *E. coli* colonies from the environment were determined by using Etest® for enrofloxacin (Biomèrieux) according to the manufacturer’s instructions.

After assigning individual *E. coli* isolates to strains by macrorestriction analysis, broth microdilution method in accordance to CLSI was used to determine MIC values against enrofloxacin and nalidixic acid. Selected *E. coli* strains, which occurred repeatedly at various points in time over the duration of the experiments in the same animal, or which were found in both groups and/or in the environment, were monitored. Therefore, MIC_90_-values were used for evaluation.

### Macrorestriction analysis

To monitor the spread and the distribution of individual *E. coli* strains pulsed-field gel electrophoresis (PFGE) was used. *E. coli* isolated from Endo agar without supplementation of enrofloxacin from two randomly chosen pigs per group as well as selected isolates from Endo agar with supplementation of 0.125 mg/L and 4 mg/L enrofloxacin were chosen for macrorestriction analysis as previously described [37]. Bacterial DNA was digested with the restriction enzyme XbaI. 100 μM thiourea were added to the running buffer (0.5XTBE) to avoid DNA degradation by Tris radicals [38]. DNA fragment patterns were examined by ethidium bromide staining and compared using the software platform BioNumerics (version 7.1, Applied Maths, Sint-Martens-Latem, Belgium).

### Molecular characterisation by PCR

One representative isolate of each *E. coli* strain was tested for the occurrence of virulence genes, adhesion gene and genes for iron acquisition. The selected genes are shown in Table 3. PCRs were performed as described previously [39, 40]. Classification according to the *E. coli* Reference Collection (EcoR) system was based on the rapid phylogenetic grouping PCR technique described by Clermont et al. [41]. Isolates were assigned to one of four groups (A, B1, B2 or D). Genes for plasmid-mediated fluoroquinolone resistance (*qnrA, qnrB* and *qnrS*) were detected as described by Robicsek et al. [19].

### Enro- and ciprofloxacin in sedimentation samples

Sedimentation samples of dust from the stable were collected at four different localisations in the stable before (Fig. 1), during and after both administration periods, respectively. For that, two Makrolon® panels with 0.16 m^2^ surface each were respectively placed on the alley in front of each feeding trough of both groups, on the opposite alley between both groups and apart from the animals in the working area of the stable, to serve as surface for the sedimentation of dust. The Makrolon® panels were placed five days before administration started to ensure a sufficient amount of sedimented dust to collect for analysis. The extraction of enrofloxacin and its metabolite ciprofloxacin as well as the analysis performed by using high-performance liquid chromatography with fluorescence detection was carried out as described by Scherz [42].

### Definitions and statistical analysis

Each coliform colony picked from a plate and identified as *E. coli* was defined as an *E. coli* isolate. *E. coli* isolates with MIC values against enrofloxacin above the epidemiological cut-off value (ECOFF) of 0.125 mg/L were defined as N-WT isolates.

Isolates with the same specific macrorestriction enzyme pattern were assigned to one *E. coli* strain. The diversity of *E. coli* strains was measured as defined by Katouli et al. [43] with Simpson's index of diversity (Di) [44]. *E. coli* strains detected from at least two of three potential sources (experimental group, control group, environment) were described as transferable strains.

Statistical evaluations were performed by the software GraphPad Prism software (GraphPad Prism®5.01, Graph Pad Software, San Diego, CA, USA) and SAS (SAS®9.3 Software, SAS, Cary, NC, USA). Medians of MIC values from *E. coli* isolates of experimental and control group on each trial day were compared with the non-parametric Wilcoxon test for independent samples. Fisher's exact test was used to calculate significant differences between the occurrence of single genes and the phylogenetic affiliation of individual *E. coli* strains. Correlation between frequency and transferability of detected *E. coli* strains and single genes were tested by Spearman correlation coefficient. Results were considered statistically significant at a significance level of α < 0.05.

## Results

### Number of *E. coli* isolates

Throughout the sampling period, a total number of 1444 *E. coli* isolates were obtained from the experimental group (EG) (748 isolates), the control group (CG) (508 isolates) and the environment (En) (188 isolates). Further information is shown in Table 1.

**Table 1:** Number of *E. coli* isolates cultured included in further analyses

	Enrofloxacin concentration of Endo agar plates			∑
	0 mg/L	0.125 mg/L	4 mg/L	
EG	361	267	120	748
CG	423	55	30	508
En	188	n.t.	n.t.	188
∑	972	322	150	1444

*EG* experimental group, *CG* control group, *En* environment

On Endo agar plates without supplementation of enrofloxacin, coliform growth occurred with each sampling in the control group and in the environment. Within the experimental group limited or no coliform growth was shown always one day after administration of enrofloxacin. Therefore, it was not possible to obtain ten isolates from all pigs one day after administration.

On Endo agar plates supplemented with enrofloxacin, coliform growth was found earlier and more often in the experimental group, where first isolates above the ECOFF could be detected on day 11 (Endo agar with 0.125 mg/L enrofloxacin) and on day 35 (Endo agar with 4 mg/L enrofloxacin). Within the control group first coliform growth occurred on day 28 (Endo agar with 0.125 mg/L enrofloxacin) and on day 42 (Endo agar with 4 mg/L enrofloxacin).

### Enrofloxacin susceptibility of isolated *E. coli*

**Fig. 2:**
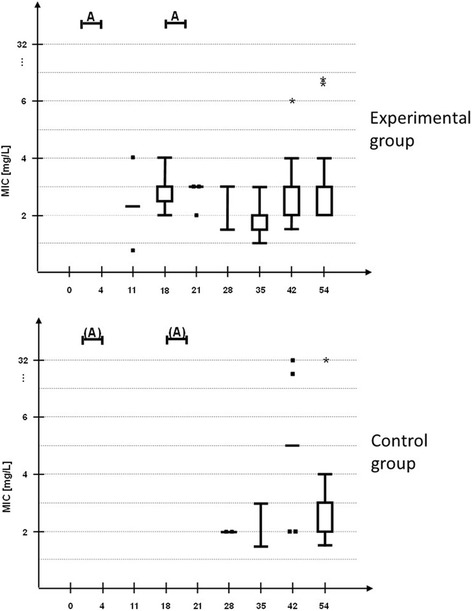
Distribution of MIC values of *E. coli* isolates. MIC values were determined by Etest® during the experimental trial in the experimental group (EG) (n=6 pigs) compared to the control group (CG) (n=6 pigs) housed in the same stable. MIC values are represented in Tukey boxplots, points represent single MIC-values, asterisks outliers. Up to 10 *E. coli* colonies per animal and day were isolated from Endo agar plates supplemented with 0.125 mg/L enrofloxacin to screen for MIC-shifts (total number of isolated *E. coli* colonies per day represented in brackets). A= intramuscular administration of EG with 2.5 mg/kg bw for three days

Susceptibility shifts of commensal *E. coli* isolates were determined in both groups at various points in time of the trial as shown in Fig. 2. Only susceptible wild type (WT)-*E. coli* isolates with MIC values below the ECOFF were found before starting the experimental trial. One week after the first administration period (day 11), N-WT-*E. coli* with MIC values above the ECOFF could be cultivated from faecal samples of two animals from the experimental group for the first time. After the second administration period faecal samples from all pigs of the experimental group harboured N-WT-*E. coli*. N-WT-*E. coli* in faecal samples of the control group could be detected for the first time seven days after the second administration period on day 28. Only one out of six animals was affected. At the end of the trial *E. coli* with MIC values above the ECOFF were determined in faecal samples from all animals of the control group. *E. coli* isolates with MIC values > 4 mg/L were determined in both groups about three weeks after the second administration period. Environmental N-WT-*E. coli* isolates could be isolated for the first time on day 32, ten days later one *E. coli* isolate with a MIC value above 4 mg/L was detected.

Significant differences between MIC values from *E. coli* isolates of the experimental and the control group could not be detected at no trial day.

### Time-dependent occurrence of *E. coli* strain pattern

The total number of *E. coli* isolates selected for macrorestriction analysis was 429. Among these isolates, we identified 64 different pulsed-Field Gel Electrophoresis (PFGE) strains with a mean occurrence of 6.7 times (range 1 to 64). More than 50% of the strains could not be detected more than twice, whereas 23.4% occurred more than ten times. Further information on the distribution of *E. coli* strains can be found in Table 2.

**Table 2:** Occurrence of *E. coli* PFGE strains during the whole trial

Total number of different PFGE type from this source:	Distribution within all sources				
	Only in this group	Coexistent in En	Coexistent in CG	Coexistent in EG	In all groups
En: *n*=20	7		3	3	7
CG: *n*=44	20	3		14	
EG: *n*=34	10	3	14		

*EG* experimental group, *CG* control group, *En* environment, *PFGE* Pulsed-Field Gel Electrophoresis

27 of the detected strains (42.2%) were transferred between at least two of the three potential sources (experimental group, control group, environment). Few strains could be detected throughout the entire experimental period; most strains were seen once or for up to two weeks.

Changes of the composition of *E. coli* population of two pigs, which were randomly chosen from each group, were determined. DNA fragment patterns of fifty *E. coli* isolates from each pig (10 isolates per trial day) were analysed. Before administration, diversity of *E. coli* strains varied strongly regardless of pig’s group affiliation (diversity indices (Di) between 0.6 and 0.93). Directly after each administration, almost no *E. coli* colony could be isolated from the two pigs of the experimental group, resulting in a diversity index close to zero. One week after each administration period diversity indices of *E. coli* isolates from both pigs of the experimental group varied strongly (0.2 vs 0.76), whereas the diversity of *E. coli* from the control group was still high (Di ≥ 0.84). On day 54 diversity of *E. coli* strains from both pigs of the experimental group and one pig of the control group was high (Di ≥ 0.83). However, *E. coli* from the other pig of the control group showed lower diversity (Di = 0.38). Overall diversity of *E. coli* strains from each individual pig varied uniquely during the whole trial regardless of administration or no administration.

### Changes of individual *E. coli* strains regarding their susceptibilities during the experimental trial

In total, 25 *E. coli* strains could be detected repeatedly during the whole trial isolated from at least two of three potential sources. MIC determination was done for at least two isolates of the same strain. MIC changes of more than two titre steps were assumed as a relevant shift in susceptibility behaviour.

**Fig. 3:**
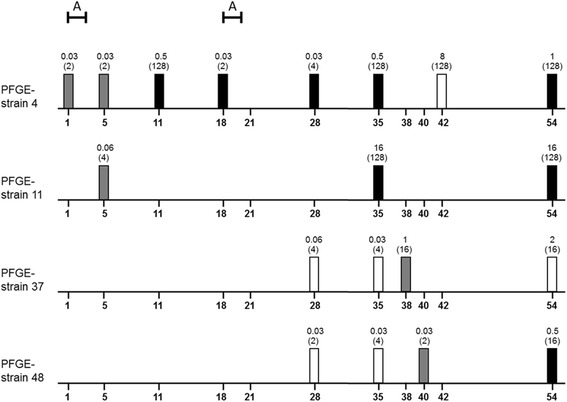
Distribution of four *E. coli* PFGE strains with susceptibilities shifts against fluoroquinolones. The X-axis represents the trial period (in days). The columns show the origin of *E. coli* strains (white: CG, grey: environment, black: EG). The data above the columns are MIC values against enrofloxacin and nalidixic acid (in brackets). A=administration period

An increase in MIC values specifically against fluoroquinolones was found in four strains. These results are presented in Fig. 3.

### Molecular characterisation of *E. coli* strains by PCR

Phylogenetic affiliation and presence of virulence genes, adhesion genes and genes for iron acquisition of 64 *E. coli* strains are listed in Table 3. Most strains belonged to EcoR group A (71.9% of isolates) followed by groups B1 (21.9%), D (4.7%) and B2 (1.5%). Virulence genes were not detected in any strain. The genes *crlA* and *fimC* were present in more than three-quarters of all isolated *E. coli* strains. Other genes related with adhesion or iron acquisition occurred only sporadically. No strain harboured the adhesion related gene *afa/draB*. We also screened the strains for plasmid-mediated horizontally transferable genes encoding quinolone resistance: 12 *E. coli* strains were at least temporarily positive for *qnrS.* Further information is given in Table 4 *. QnrA* and *qnrB* could not be detected in any strain.

**Table 3:** Prevalence of virulence, adhesion, and iron acquisition genes in 64 intestinal *E. coli* strains

Gene or operon	Description	Total Prevalence [%]
Virulence gene		
*stx1*	shigatoxin 1	0
*stx2*	shigatoxin 1	0
*eae*	intimin	0
*sta*	heat-stable enterotoxin	0
*F41*	fimbriae F41	0
*K99*	fimbriae K99	0
Adhesins		
*afa/draB*	Afimbrial/Dr antigen-specific adhesion	0
*crlA*	curli fibre gene	92.2
*fimC*	Type 1 fimbriae	78.1
*hra*	Heat-resistant agglutinin	6.3
*papC*	Pilus associated with pyelonephritis	1.6
*sfa/foc*	S fimbriae and F1C fimbriae	1.6
*tsh*	Temperature-sensitive haemagglutinin	3.1
Iron acquisition		
*chuA*	Heme receptor gene	6.3
*fyuA*	Ferric yersinia uptake	10.9
*ireA*	Iron-responsive element	1.6
*iroN*	Catecholate siderphore	3.1
*iucD*	Aerobactin synthesis	4.7
*sitD chr.*	*Salmonella* iron transport system gene	1.6
*sitD ep.*	*Salmonella* iron transport system gene	4.7

**Table 4:** Occurrence of *E. coli* PFGE strains with *qnrS*

PFGE strain	Also detected without *qnrS*	First detection		MIC ENRO [mg/L]	ECOR group
		Source	Day		
I-12	yes	EG (CG)^a^	35 (11)	0.03 (0.06)	A
I-21	no	EG	54	1	A
I-25	yes	CG (En)	42 (5)	8 (0.03)	A
I-28	no	EG	54	1	A
I-31	no	CG	54	1	B1
I-34	no	EG	21	1	B1
I-37	yes	EG (EG)	54 (54)	1 (0.03)	A
I-38	yes	CG (CG)	54 (18)	2 (n.t.)	A
I-41	yes	EG (CG)	54 (21)	1 (n.t.)	A
I-45	no	EG	54	1	A
I-51	yes	EG (CG)	54 (11)	1 (n.t.)	A
I-56	no	CG	54	1	A

*EG* experimental group, *CG* control group, *En* environment, *PFGE* Pulsed-Field Gel Electrophoresis, *n.t.* not tested, *ENRO* Enrofloxacin

^a^Number in brackets apply to the same PFGE strain without *qnrS*

Significant associations between occurrence, frequency and transferability of detected *E. coli* strains and the occurrence of single genes as well as their phylogenetic affiliation were not detected.

### Environmental load with enrofloxacin and ciprofloxacin

**Fig. 4:**
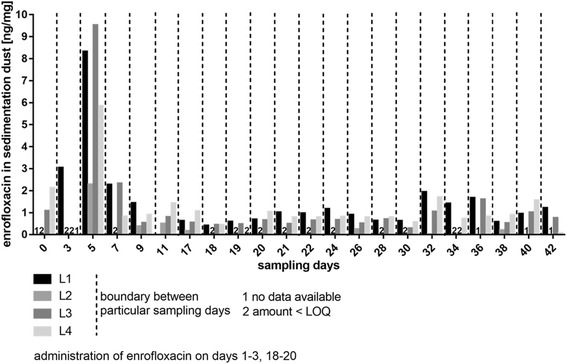
Distribution of enrofloxacin in sedimentation dust in dependence of the location of sampling. L1-4: Location 1 to 4 of sedimentation dust sampling. For orientation make use of the schematic representation of the stable (Fig. 1)

As shown in Fig. 4 the highest amounts of enrofloxacin were found in front of the feeding trough of the experimental group. The environmental load with enrofloxacin in sedimentation dust increased during the first administration period and showed the highest amount of the antibiotic during the first administration and two days afterwards. At that time the concentration ranged between 5 and 10 ng enrofloxacin/mg sedimentation dust at the different locations. The more time passed, the more sedimentation dust accumulated and minimal quantities between 0 and 2 ng enrofloxacin/mg sedimentation dust were detectable.

Only traces of the active metabolite ciprofloxacin could be found during the experimental trial.

## Discussion

Each use of antibiotics increases the risk of antimicrobial resistance [45, 46]. Besides, several studies have shown that antimicrobial use in food producing animals leads to increasing resistance levels in both pathogenic and commensal bacteria [47]. Many recent studies also demonstrated the increased risk of AMR in commensal *E. coli* after oral administration of antibiotics compared to untreated pigs [48–50]. However, there is a lack of studies comparing the impact of the route of administration on the development of resistance.

In this study we focused as a first step on changes in the intestinal commensal *E. coli* population after parenteral administration of pigs with fluoroquinolones as a potential and approved alternative to oral medication. Since we could not detect any *E. coli* strain during the whole experimental trial showing virulence genes typical for porcine enteropathogenic *E. coli*, it is likely that only commensal intestinal *E. coli* were included in this study.

The parenteral administration of enrofloxacin at the recommended dose (2.5 mg/kg bw) to piglets considerably reduced the number of the susceptible intestinal *E. coli* population. After a few days a rising number of *E. coli* with increased MIC values against enrofloxacin could be detected. These findings are in accordance with other studies regarding the administration of fluoroquinolones [27, 51, 52].

There are two different types of criteria to interpret MIC values: clinical breakpoints and ECOFFs [53]. For *E. coli* from chicken and turkeys the Clinical Laboratory Standard Institute (CLSI) has determined a veterinary specific breakpoint of ≥ 2 mg/L enrofloxacin [54]. A veterinary specific breakpoint for enrofloxacin is not available for Enterobacteriaceae in pigs. By ECOFFs bacterial populations are divided into a wild type (WT) population without any acquired resistance mechanisms and a N-WT population with higher MIC values or smaller zone diameters [53]. To evaluate enrofloxacin susceptibilities of isolated *E. coli* we used the ECOFF (0.125 mg/L) from the European Committee on Antimicrobial Susceptibility Testing [33].

*E. coli* isolates with MIC values above the enrofloxacin ECOFF were observed one week after the first administration period for the first time. This result was in agreement with those of Wiuff et al. [26] who described a very rapid occurrence of fluoroquinolone resistance among the porcine coliform flora during administration with enrofloxacin. As expected, increased MIC values above 0.125 mg/L enrofloxacin occurred initially in *E. coli* isolates from the experimental group with increasing detection rates until the end of the trial. Subsequently and to a lesser extent also pigs of the control group in the same pen exhibited N-WT-*E. coli*. In similar studies, it has been shown that ceftiofur resistant *E. coli* occurred in untreated pigs which were housed in the same stable like a treated pig group [55]. These findings may be due to the transferability of N-WT strains from the experimental group through the environment to the control group especially as we could isolate the same PFGE strains from both pig groups and the environment. However, a causal link between the prevalence and transferability of single *E. coli* strains and detected adhesion genes, genes for iron acquisition or phylogenetic affiliation could not be identified.

Another possible root for the detection of N-WT strains within pigs of the control group might be the exposure of WT *E. coli* strains to subtherapeutic doses of enrofloxacin. Enrofloxacin and its main and active metabolite ciprofloxacin, which is known as a broad spectrum antimicrobial in human medicine, were found in minimal concentrations in sedimentation dust in the stable. These findings indicate that *E. coli* from untreated pigs could be exposed to enrofloxacin concentrations in lower than therapeutic doses from the environment resulting in an effective selective pressure. Scherz et al. [34] showed that a long-time exposure of 21 days of the commensal flora of poultry to subtherapeutic doses of enrofloxacin leads to an amplification and selection of N-WT-*E. coli* strains, which persist in the commensal microbiota. In the present study, the concentrations of enrofloxacin vary in the lower nanogram range in sedimented dust while only traces of ciprofloxacin are detectable. Gullberg et al. [56] demonstrated for ciprofloxacin *in vitro* that amounts of 1/10 and 1/230 of the MIC of a susceptible strain can select for N-WT strains by reducing the growth rates of the susceptible ones and balancing the fitness costs of the resistant bacteria. It is unknown to what extent the minimal amounts of enro- and ciprofloxacin in sedimented dust in the present case affect the commensal flora. However, in case of carry-over *in vivo* environmental bacteria are exposed to these contaminations.

In addition, the cross contamination between both groups by veterinarians and animal care staff during feeding, administration and sampling measures cannot be completely ruled-out in spite of all preventive measures taken.

In this study, 12 of 64 PFGE strains were at least temporarily positive for *qnrS*. As we did not analyse mutations within the gyrase and topoisomerase we cannot determine whether the observed reduced fluoroquinolone susceptibilities is caused by chromosomal mutations, by PMQR or a combination of both. But it is assumed that especial *qnr* mediated resistance results in fluoroquinolone resistance to a limited extent and leads to slightly elevated MIC values [19, 57]. Nevertheless it is horizontally transferable in Enterobacteriaceae and thereby contributes to the wide spread of reduced fluoroquinolone susceptibility.

To prevent selection of chromosomal mutations resulting in reduced susceptibility to fluoroquinolones, the dose regimen should be based on pharmacokinetics parameters in both plasma and target tissues. The mutant prevention concentration (MPC) is the minimum concentration preventing growth at a high inoculum (≥10^9^ CFU/mL) using agar dilution methodology [58]. Drug levels in target tissues above the MPC are necessary to severely restrict a first step mutation as far as concentration dependent antibiotics like fluoroquinolones are concerned [59]. However, further studies are required to assess the clinical significance of the MPC.

N-WT strains may also be already present in small amounts within the coliform microflora or in the environment and could have used the selective advantage during antimicrobial administration. In our study, exclusively pigs from farms without any coliform growth on Endo agar supplemented with 0.125 mg/L enrofloxacin were included. No N-WT strain could be detected in the stable and all animals within the first five days after the beginning of administration. In addition, we were able to detect the occurrence of *E. coli* strains with the same PFGE patterns showing different susceptibilities against the tested fluoroquinolones. Therefore we assume that the observed N-WT strains are based at least partially on the acquisition of resistance traits by previously completely susceptible strains and the following spread of these N-WT strains. However, our screening of the stable prior to the arrival of the pigs could not include the whole area of all pens and our sampling design only included the examination of a small amount of faeces. Therefore, we cannot totally exclude the presence of N-WT strains prior to the first administration. *E. coli* strains isolated from faeces may not be representative of *E. coli* from other regions of the gastrointestinal tract [60]. Therefore, further studies are necessary which include the coliform flora colonizing the lower intestine (jejunum, ileum and colon).

## Conclusions

The parenteral administration of enrofloxacin at the recommended dose to piglets considerably reduced the number of the susceptible intestinal *E. coli* population which was replaced by a rising number of *E. coli* with increased MIC values. Also, pigs of the control group in the same pen without direct contact exhibited N-WT-*E. coli* suggesting the transferability of single *E. coli* strains.

Resistance in particular to critically important antimicrobials is a significant public health threat as it limits the number of effective antimicrobial agents available for therapy. The constant excretion of N-WT strains within more than four weeks after the second administration and the putative transmission of N-WT *E. coli* strains to pigs of the control group or to the environment may contribute directly to the spread of resistant bacteria to farm workers or slaughterhouse staff. Furthermore, the introduction of N-WT bacteria and resistance determinants into the food chain or the spreading of animal-by-products such as manure and slurry harbouring high levels of potentially N-WT bacteria may facilitate the exchange of resistance determinants between livestock and humans.

## Abbreviation

°C: degree Celsius
μL: microliter
AMR: Antimicrobial resistance
bw: bodyweight
CFU: Colony-forming units
CG: Control group
CLSI: Clinical and Laboratory Standards Institute
Di: Simpson's index of diversity
DNA: Deoxyribonucleic acid
*E. coli*: *Escherichia coli*
e.g.: exempli gratia
ECOFF: Epidemiological cut-off value
EcoR: *E. coli* reference collection
EG: Experimental group
En: environment
EUCAST: European Committee on Antimicrobial Susceptibility Testing
HPCIA: Highest priority critically important antimicrobials
HPLC: High performance liquid chromatography
i.m.: intramuscular
kg: kilogram
LB: Luria-Bertani Bouillon
mg/L: milligrams per Litre
MIC: Minimum inhibitory concentration
mL: millilitre
MPC: Mutant prevention concentration
ng: nanogram
N-WT: Non-Wild Type, in this context: bacterial populations with acquired resistance mechanisms resulting in decreased susceptibilities to fluoroquinolones
OIE: World Organisation for Animal Health
PCR: Polymerase chain reaction
PFGE: Pulsed-field gel electrophoresis
PMQR: Plasmid mediated Quinolone Resistance
WHO: World Health Organization
WT: Wild Type, in this context: bacterial populations without any acquired resistance mechanisms against fluoroquinolones

## References

[1] Lee CR, Cho IH, Jeong BC, Lee SH (2013). Strategies to minimize antibiotic resistance. Int J Environ Res Public Health..

[2] Mazurek J, Pusz P, Bok E (2013). The phenotypic and genotypic characteristics of antibiotic resistance in *Escherichia coli* populations isolated from farm animals with different exposure to antimicrobial agents. Pol J Microbiol..

[3] Volkova VV, Lanzas C, Lu Z, Gröhn YT (2012). Mathematical model of plasmid-mediated resistance to ceftiofur in commensal enteric *Escherichia coli* of cattle. PLoS One..

[4] Chantziaras I, Boyen F, Callens B, Dewulf J (2014). Correlation between veterinary antimicrobial use and antimicrobial resistance in food-producing animals: a report on seven countries. J Antimicrob Chemother.

[5] Rodríguez-Martínez JM, Cano ME, Velasco C (2011). Plasmid-mediated quinolone resistance: an update. J Infect Chemother..

[6] World Health Organization (WHO), WHO Advisory Group on Integrated Surveillance of Antimicrobial Resistance (AGISAR): Critically important antimicrobials for human medicine – 3rd rev. http://www.who.int (2011). Accessed 18 Nov 2016.

[7] World Organization for Animal Health (OIE): OIE List of antimicrobials of veterinary importance. http://www.oie.int (2007). Accessed 18 Nov 2016

[8] Appelbaum PC, Hunter PA (2000). The fluoroquinolone antibacterials: past, present and future perspectives. Int J Antimicrob Agents..

[9] Bugyei K, Black WD, Ewen S (1999). Pharmacokinetics of enrofloxacin given by the oral, intravenous and intramuscular routes in broiler chickens. Can J Vet Res..

[10] Brown SA (1996). Fluoroquinolones in animal health. J Vet Pharmacol Ther..

[11] Scheer M (1987). Concentrations of active ingredient in serum and in tissues after oral and parenteral administration of Baytril. Vet. Med. Rev..

[12] Wiuff C, Lykkesfeldt J, Aarestrup FM, Svendsen O (2002). Distribution of enrofloxacin in intestinal tissue and contents of healthy pigs after oral and intramuscular administrations. J Vet Pharmacol Ther..

[13] Lode H, Borner K, Koeppe P (1998). Pharmacodynamics of fluoroquinolones. Clin Infect Dis..

[14] Drlica K, Zhao XDNA (1997). gyrase, topoisomerase IV, and the 4-quinolones. Microbiol Mol Biol Rev..

[15] Hopkins KL, Davies RH, Threlfall EJ (2005). Mechanisms of quinolone resistance in *Escherichia coli* and *Salmonella*: recent developments. Int J Antimicrob Agents..

[16] Chenia HY, Pillay B, Pillay D (2006). Analysis of the mechanisms of fluoroquinolone resistance in urinary tract pathogens. J Antimicrob Chemother..

[17] Veldman K, Cavaco LM, Mevius D (2011). International collaborative study on the occurrence of PMQR in *Salmonella enterica* and *Escherichia coli* isolated from animals, humans, food and the environment in 13 European countries. J Antimicrob Chemother..

[18] Robicsek A, Jacoby GA, Hooper DC (2006). The worldwide emergence of plasmid-mediated quinolone resistance. Lancet Infect Dis..

[19] Martínez-Martínez L, Pascual A, Jacoby GA (1998). Quinolone resistance from a transferable plasmid. Lancet..

[20] Jacoby GA (2005). Mechanisms of resistance to quinolones. Clin Infect Dis..

[21] Devreese M, Antonissen G, De Baere S, De Backer P, Croubels S (2014). Effect of administration route and dose escalation on plasma and intestinal concentrations of enrofloxacin and ciprofloxacin in broiler chickens. BMC Vet Res..

[22] European Medicines Agency (EMA): Opinion following an Article 35 referral for Baytril 2.5% injectable, Baytril 5% injectable and Baytril 10% injectable and their associated names, and related veterinary medicinal products - Background information (EMEA/V/A/097). http://www.ema.europa.eu (2014). Accessed 18 Nov 2016.

[23] Robanus M, Hegger-Gravenhorst C, Mollenhauer Y, Hajek P, Käsbohrer A, Honscha W, Kreienbrock L (2014). Feasibility study of veterinary antibiotic consumption in Germany - comparison of ADDs and UDDs by animal production type, antimicrobial class and indication. BMC Vet Res..

[24] European Food and Safety Agency and (2014). (EFSA) and European Food Safety Authority and European Centre for Disease Prevention and Control (ECDC): The Community Summary Report on antimicrobial resistance in zoonotic and indicator bacteria from humans, animals and food in 2012. EFSA Journal..

[25] Pereira RV, Siler JD, Ng JC, Davis MA, Grohn YT, Warnick LD (2014). Effect of on-farm use of antimicrobial drugs on resistance in fecal *Escherichia coli* of preweaned dairy calves. J Dairy Sci..

[26] Wiuff C, Lykkesfeldt J, Svendsen O, Aarestrup FM (2003). The effects of oral and intramuscular administration and dose escalation of enrofloxacin on the selection of quinolone resistance among *Salmonella* and coliforms in pigs. Res Vet Sci..

[27] Walker RD, Prescott JF, Baggot JD, Walker RD (2000). Fluoroquinolones. Antimicrobial Therapy in Veterinary Medicine. 3.

[28] Fadário Frade VM, Dias M, Costa Teixeira ACS, Alves Palma MS. Environmental contamination by fluoroquinolones. Braz. J Pharm Sci. 2014;50:1.

[29] Douglas R. Call, Louise Matthews, Murugan Subbiah, Jinxin Liu, (2013) Do antibiotic residues in soils play a role in amplification and transmission of antibiotic resistant bacteria in cattle populations?. Frontiers in Microbiology 4. 10.3389/fmicb.2013.00193 PMC3708158 23874327

[30] Bertani G (1951). Studies on lysogenesis. I. The mode of phage liberation by lysogenic *Escherichia coli*. J. Bacteriol..

[31] Atlas RM, Snyder JW (2006). Handbook of Media for Clinical Microbiology.

[32] The European Committee on Antimicrobial Susceptibility Testing (EUCAST): MIC distributions and ECOFFs. http://www.eucast.org/mic_distributions_and_ecoffs (2016). Accessed 18 Nov 2016.

[33] Corry JEL, Curtis GDW, Baird RM (2012). Handbook of culture media for food and water Microbiology.

[34] Scherz G, Stahl J, Glünder G, Kietzmann M (2014). Effects of carry-over of fluoroquinolones on the susceptibility of commensal *Escherichia coli* in the intestinal microbiota of poultry. Berl Munch Tierarztl Wochenschr.

[35] Edén CS, Eriksson B, Hanson LA (1978). Adhesion to normal human uroepithelial cells of *Escherichia coli* from children with various forms of urinary tract infection. J Pediatr..

[36] Döpfer D, Buist W, Soyer Y (2008). Assessing Genetic Heterogeneity within Bacterial Species Isolated from Gastrointestinal and Environmental Samples: How Many Isolates Does It Take?. Appl Environ Microbiol..

[37] Schierack P, Roemer A, Jores J (2009). Isolation and characterization of intestinal *Escherichia coli* clones from wild boars in Germany. Appl Environ Microbiol..

[38] Liesegang A, Tschäpe H (2002). Modified pulsed-field gel electrophoresis method for DNA degradation-sensitive *Salmonella enterica* and *Escherichia coli* strains. Int J Med Microbiol..

[39] Ewers C, Li G, Wilking H (2007). Avian pathogenic, uropathogenic, and newborn meningitis-causing *Escherichia coli*: how closely related are they?. Int J Med Microbiol..

[40] Franck SM, Bosworth BT, Moon HW (1998). Multiplex PCR for enterotoxigenic, attaching and effacing, and Shiga toxin-producing *Escherichia coli* strains from calves. J Clin Microbiol..

[41] Clermont O, Bonacorsi S, Bingen E (2000). Rapid and simple determination of the *Escherichia coli* phylogenetic group. Appl Environ Microbiol..

[42] Scherz G. Carryover of subtherapeutic antimicrobial dosages of enrofloxacin and the influence on the development of antibiotic resistance of commensal *Escherichia coli* in the intestine of poultry (Ph.D. Thesis). University of Veterinary Medicine Hannover, Foundation; 2013.

[43] Katouli M, Lund A, Wallgren P (1995). Phenotypic characterization of intestinal *Escherichia coli* of pigs during suckling, postweaning, and fattening periods. Appl Environ Microbiol..

[44] Simpson EH (1949). Measurement of diversity. Nature..

[45] Davies J, Davies D (2010). Origins and Evolution of Antibiotic Resistance. Microbiol Mol Biol Rev..

[46] Dunlop RH, McEwen SA, Meek AH (1998). Associations among antimicrobial drug treatments and antimicrobial resistance of fecal *Escherichia coli* of swine on 34 farrow-to-finish farms in Ontario, Canada. Prev Vet Med..

[47] Van den Bogaard AE, Stobberingh EE (2000). Epidemiology of resistance to antibiotics. Links between animals and humans. Int J Antimicrob Agents..

[48] Lutz EA, McCarty MJ, Mollenkopf DF (2011). Ceftiofur use in finishing swine barns and the recovery of fecal *Escherichia coli* or *Salmonella* spp. resistant to ceftriaxone. Foodborne Pathog Dis..

[49] Varga C, Rajić A, McFall ME (2009). Associations among antimicrobial use and antimicrobial resistance of *Salmonella spp*. isolates from 60 Alberta finishing swine farms. Foodborne Pathog Dis..

[50] Wagner BA, Straw BE, Fedorka-Cray PJ, Dargatz DA (2008). Effect of antimicrobial dosage regimen on *Salmonella* and *Escherichia coli* isolates from feeder swine. Appl Environ Microbiol..

[51] Lastours de V, Cambau E, Guillard T (2012). Diversity of Individual Dynamic Patterns of Emergence of Resistance to Quinolones in Escherichia coli From the Fecal Flora of Healthy Volunteers Exposed to Ciprofloxacin. J Infect Dis..

[52] Fantin B, Duval X, Massias L (2009). Ciprofloxacin Dosage and Emergence of Resistance in Human Commensal Bacteria. J Infect Dis..

[53] Schwarz S, Kadlec K, Silley P (2013). Enteric infection. Antimicrobial resistance in bacteria of animal origin.

[54] Clinical and Laboratory Standards Institute (CLSI). Performance Standards for Antimicrobial Disk and Dilution Susceptibility Tests for Bacteria Isolated From Animals, Third Informational Supplement VET01S. Wayne, PA; 2015.

[55] Beyer A, Baumann S, Scherz G (2015). Effects of ceftiofur treatment on the susceptibility of commensal porcine *E. coli* – comparison between treated and untreated animals housed in the same stable. BMC Vet Res..

[56] Gullberg E, Cao S, Berg OG (2011). Selection of resistant bacteria at very low antibiotic concentrations. PLoS Pathog..

[57] Yue L, Jiang HX, Liao XP, Liu JH, Li SJ, Chen XY, Chen CX, Lü DH, Liu YH (2008). Prevalence of plasmid-mediated quinolone resistance *qnr* genes in poultry and swine clinical isolates of *Escherichia coli*. Vet Microbiol..

[58] Mouton JW, Vinks AA (2005). PK-PD modelling of antibiotics in vitro and in vivo using bacterial growth and kill kinetics: the MIC vs stationary concentrations. Clin Pharmacokinet..

[59] Wang J, Hao H, Huang L (2016). Pharmacokinetic and Pharmacodynamic Integration and Modeling of Enrofloxacin in Swine for *Escherichia coli*. Front Microbiol..

[60] Dixit SM, Gordon DM, Wu X (2004). Diversity analysis of commensal porcine *Escherichia coli* – associations between genotypes and habitat in the porcine gastrointestinal tract. Microbiol..

